# Influence of platelet storage time on human platelet lysates and platelet lysate-expanded mesenchymal stromal cells for bone tissue engineering

**DOI:** 10.1186/s13287-020-01863-9

**Published:** 2020-09-23

**Authors:** Siddharth Shanbhag, Samih Mohamed-Ahmed, Turid Helen Felli Lunde, Salwa Suliman, Anne Isine Bolstad, Tor Hervig, Kamal Mustafa

**Affiliations:** 1 Department of Clinical Dentistry, Faculty of Medicine, University of Bergen, Årstadveien 19, 5008 Bergen, Norway; 2 Department of Immunology and Transfusion Medicine, Haukeland University Hospital, Bergen, Norway; 3 Laboratory of Immunology and Transfusion Medicine, Haugesund Hospital, Fonna Health Trust, Haugesund, Norway; 4 Department of Clinical Science, University of Bergen, Bergen, Norway

**Keywords:** Platelet lysate, Mesenchymal stromal cells, Bone tissue engineering, Regenerative medicine

## Abstract

**Background:**

Human platelet lysate (HPL) is emerging as the preferred xeno-free supplement for the expansion of mesenchymal stromal cells (MSCs) for bone tissue engineering (BTE) applications. Due to a growing demand, the need for standardization and scaling-up of HPL has been highlighted. However, the optimal storage time of the source material, i.e., outdated platelet concentrates (PCs), remains to be determined. The present study aimed to determine the optimal storage time of PCs in terms of the cytokine content and biological efficacy of HPL.

**Methods:**

Donor-matched bone marrow (BMSCs) and adipose-derived MSCs (ASCs) expanded in HPL or fetal bovine serum (FBS) were characterized based on in vitro proliferation, immunophenotype, and multi-lineage differentiation. Osteogenic differentiation was assessed at early (gene expression), intermediate [alkaline phosphatase (ALP) activity], and terminal stages (mineralization). Using a multiplex immunoassay, the cytokine contents of HPLs produced from PCs stored for 1–9 months were screened and a preliminary threshold of 4 months was identified. Next, HPLs were produced from PCs stored for controlled durations of 0, 1, 2, 3, and 4 months, and their efficacy was compared in terms of cytokine content and BMSCs’ proliferation and osteogenic differentiation.

**Results:**

BMSCs and ASCs in both HPL and FBS demonstrated a characteristic immunophenotype and multi-lineage differentiation; osteogenic differentiation of BMSCs and ASCs was significantly enhanced in HPL vs. FBS. Multiplex network analysis of HPL revealed several interacting growth factors, chemokines, and inflammatory cytokines. Notably, stem cell growth factor (SCGF) was detected in high concentrations. A majority of cytokines were elevated in HPLs produced from PCs stored for ≤ 4 months vs. > 4 months. However, no further differences in PC storage times between 0 and 4 months were identified in terms of HPLs’ cytokine content or their effects on the proliferation, ALP activity, and mineralization of BMSCs from multiple donors.

**Conclusions:**

MSCs expanded in HPL demonstrate enhanced osteogenic differentiation, albeit with considerable donor variation. HPLs produced from outdated PCs stored for up to 4 months efficiently supported the proliferation and osteogenic differentiation of MSCs. These findings may facilitate the standardization and scaling-up of HPL from outdated PCs for BTE applications.

## Background

Adult mesenchymal stromal cells (MSCs) from various tissue sources, most frequently bone marrow (BMSCs) and adipose tissue (ASCs), are increasingly being used in bone tissue engineering (BTE) strategies for reconstruction of clinically challenging bone defects [1]. Although the use of whole tissue fractions, such as bone marrow concentrates and adipose stromal vascular fractions (SVFs), offers the feasibility of minimum cell manipulation and cost-effectiveness, the yield of MSCs obtained is relatively low. MSCs represent < 1% of the mononuclear cell fraction in the bone marrow and approximately 1.4% in adipose SVF [2]. This has encouraged ex vivo expansion strategies, which aim to exponentially amplify the number of BMSCs or ASCs available for implantation and thereby improve clinical outcomes.

The use of safe, standardized, and efficacious culture conditions is a critical aspect of Good Manufacturing Practice (GMP)-grade MSC expansion. Supplements providing growth factors (GFs), proteins, and enzymes for ex vivo MSC expansion are broadly categorized as xenogeneic (animal-derived), xeno-free (human-derived), or chemically defined [3, 4]. Although fetal bovine serum (FBS) is commonly used for MSC expansion [5], several limitations of FBS supplementation have been highlighted [3, 6]. European guidelines advocate the use of “non-ruminant” over “ruminant materials” for the manufacture of human medicinal products [7]. Accordingly, an increase in the use of “xeno-free” supplements, such as human platelet lysate (HPL), to develop GMP-compliant MSC expansion protocols has recently been reported [4, 8].

HPL is defined as a cell-free, protein- and GF-rich biological material produced from platelet concentrates (PCs) initially intended for transfusion [9]. Platelets release a wide range of physiological GFs and cytokines, which can significantly enhance cell growth and function. Pooled- and/or single-donor apheresis PCs are routinely prepared by blood establishments for transfusion and, depending on local regulations, stored for a maximum of 4–7 days before being discarded [9]. It is estimated that 5–20% of PCs produced in transfusion centers become “outdated” and utilizing these for HPL production is reported to be an ethically and economically optimal strategy, due to comparable efficacy of HPL produced from “fresh” and outdated PCs [6]. The current literature consistently demonstrates that HPL is at least comparable, and often superior, to FBS in supporting MSC proliferation, stromal phenotype, chromosomal stability, and multi-lineage differentiation potential [10]. Interestingly, MSCs expanded in HPL have been reported to demonstrate enhanced osteoblastic differentiation potential, suggesting particular benefits of HPL expansion for BTE applications [4]. A clinically validated protocol for MSC expansion in HPL for BTE applications has recently been published [11].

The importance of HPL in GMP-grade MSC production is highlighted by the publication of several recent consensus statements [9, 12–14]. The most common themes in these reports are the need to scale-up HPL production by blood establishments and, more urgently, the need for standardization of HPL products. There is currently considerable large variation in the methods used to produce HPL, which is further complicated by the availability of several inadequately defined commercial HPL products. A need for standardization has been described at various levels of the HPL production process, such as the source material (pooled buffy coats vs. apheresis PCs and fresh vs. outdated PCs) and storage medium [plasma vs. platelet additive solution (PAS) or a combination]. Moreover, the pool sizes, i.e., the number of PC units or individual donations that are pooled to produce a single HPL product, method of platelet lysis, use of pathogen inactivation strategies, and quality control/release criteria for the final product vary between manufacturers [14].

Nevertheless, there is a clear consensus that the use of outdated pooled PCs as the source material is the optimal strategy for large-scale HPL production. Although the storage time of PCs varies between blood centers based on national regulations, recent recommendations call for immediate freezing of outdated PCs, i.e., within 7 days after collection, for subsequent HPL production—this represents an efficient use of resources and minimizes waste [9]. However, for many blood centers, it may not always be possible to initiate HPL production on the day of (or soon after) PC expiry, and the maximum duration for which PCs can be stored before being used to prepare an efficient HPL remains unknown. If outdated PCs can be stored for a standardized period to produce an optimal HPL product, it would facilitate logistical solutions and encourage more blood establishments to incorporate HPL production into their protocols. Thus, optimizing the storage time of PCs would be a step towards addressing *both* the standardization *and* scaling-up of HPL production.

In the context of BTE, a recent study demonstrated differential effects of commercial HPL products on the mineralization capacity of BMSCs, although the mechanisms and HPL components contributing to these differences were not studied [15]. It would be of interest to investigate the effects of PC storage times on the cytokine contents of HPL, and subsequently the proliferation kinetics and osteogenic differentiation potential of HPL-expanded MSCs. Therefore, the objectives of this study were to characterize HPL in terms of its cytokine content and efficacy for MSC expansion (vs. FBS), particularly for BTE applications, and to investigate the effect of PC storage time on the cytokine content and efficacy of HPL in terms of MSC proliferation and osteogenic differentiation.

## Materials and methods

### Production of HPL

#### PC preparation and storage

The HPL herein (Bergenlys®, Bergen, Norway) is prepared from outdated pooled whole blood-derived PCs. The PCs are prepared at the Department of Immunology and Transfusion Medicine, Haukeland University Hospital, Bergen, Norway, according to established procedures and in line with national and EU quality requirements. Briefly, written informed consent is obtained from volunteer, healthy blood donors (aged 18–70 years) complying with national guidelines for blood donation. Whole blood is processed with the Reveos® Automated Blood Processing Unit (Terumo BCT, Lakewood, CO, USA). All donations are tested for ABO and RhD blood groups, infectious disease markers (HIV1/2, HBV, HCV), and sterility (aerobic bacteria). Donor information and manufacturing details are stored to ensure traceability of the final product. PCs (~ 300 mL) are generated by manually pooling five interim platelet units (IPUs) in 30% plasma and 70% platelet additive solution (Terumo BCT) and subsequently leukocyte-filtered (Immuflex®, Terumo BCT). Pooled PCs containing > 2 × 10^11^ platelets (and < 1 × 10^6^ leukocytes) are X-ray irradiated at a dose of 25 Gy and stored at 22 °C ± 2 °C under agitation for no longer than 7 days for use as transfusion units. All unused (or outdated) 7-day-old PCs are frozen at − 80 °C within 24 h for subsequent HPL production.

#### HPL production

Unused 7-day-old PCs were used for HPL production via the freeze/thaw lysis method [16]. Briefly, four different PCs (each PC containing buffy coats from five donors = 4 × 5 = 20 donors per HPL product) were exposed to multiple freezing (− 80 °C for at least 3 h) and thawing cycles [+ 37 °C in a plasma thawer (Plasmatherm®, Barkey GmbH Co. KG, Leoppoldshoehe, Germany) for 15 min] to ensure platelet lysis before pooling. Pooled PCs were then centrifuged at 3000×*g* (4 °C, 15 min) to remove platelet fragments and aliquoted as the final HPL product. No fibrinogen depletion step was performed. HPL aliquots were stored at − 80 °C and thawed overnight at 4 °C for subsequent use in experiments.

### Cell culture with HPL

#### Isolation and expansion of donor-matched BMSCs and ASCs

The biological efficacy of HPL was tested in various cellular assays using human BMSCs and ASCs. Donor-matched BMSCs and ASCs were isolated and expanded according to established protocols [17]. Briefly, human adipose tissue and bone marrow aspirates were obtained after informed parental consent and ethical approval (2013-1248/Regional Ethical Committee, South East, Norway) from patients aged 8–14 years undergoing surgery at the Department of Plastic Surgery, Haukeland University Hospital. For each donor, BMSCs and ASCs were isolated in 5% HPL and 10% FBS (GE Healthcare, South Logan, UT, USA) supplemented growth media [Dulbecco’s modified Eagle’s medium (DMEM, Invitrogen, Carlsbad, CA, USA) with 1% antibiotics (penicillin/streptomycin; GE Healthcare)]. In HPL-supplemented media, 1 IU/mL of heparin was added to prevent gelation and the medium was sterile filtered (0.2 μm) before use. Cells were sub-cultured and expanded according to a clinically validated protocol with a seeding density of 4000 cells/cm^2^ [11]; passage 2–4 cells from at least three different donors were used in experiments. Cell number and viability were assessed using 0.4% Trypan blue stain (Invitrogen) and a Countess® Automated Cell Counter (Invitrogen).

#### Immunophenotype of BMSCs and ASCs

The immunophenotype of BMSCs and ASCs in HPL and FBS was assessed by flow cytometry based on the expression of specific surface antigens, as previously described [17] according to the “minimal criteria” for defining MSCs [18]. Briefly, the cells in HPL and FBS were incubated with conjugated antibodies against selected “negative” (CD34, CD45, HLA-DR) and “positive” (CD73, CD90, CD105) MSC markers (all from BD Biosciences, San Jose, CA, USA) and STRO-1 (Santa Cruz Biotechnology, Dallas, TX, USA) following the manufacturers’ recommendations. Quantification was performed with a BD LSR Fortessa cell analyzer (BD Biosciences), and data were analyzed using flow cytometry software (FlowJo V10, Flowjo, LLC, Ashland, OR, USA).

#### Cell proliferation based on DNA quantification

BMSCs and ASCs in HPL and FBS were seeded in 24-well plates at a density of 4000 cells/cm^2^. After 1, 7, and 14 days of culture, DNA quantification was performed using the Quant-IT® PicoGreen dsDNA Assay Kit (Thermo Fisher Scientific, Carlsbad, CA, USA) according to the manufacturer’s instructions. Briefly, cells were lysed in 0.1% Triton X-100 and the PicoGreen staining solution was added and incubated for 5 min at RT protected from light, before fluorescence was measured at 480 nm (Ex)/520 nm (Em) with a microplate reader. DNA concentrations (ng/mL) were calculated based on known standards.

#### Multi-lineage differentiation of BMSCs and ASCs

The ability of BMSCs and ASCs to differentiate into multiple stromal lineages was tested as previously described [17]. Briefly, for adipogenic differentiation, cells in HPL and FBS were cultured in StemPro® adipogenic differentiation medium (Invitrogen) or standard growth medium (control). After 14 days, intracellular lipid formation was assessed via Oil red O (Sigma-Aldrich) staining. For quantification, the stain was extracted using 99% isopropanol (Sigma-Aldrich) and absorbance was measured at 540 nm using a microplate reader. For osteogenic differentiation, cells in HPL and FBS were cultured in osteogenic differentiation medium prepared by adding final concentrations of 0.05 mM L-ascorbic acid 2-phosphate, 10 nM dexamethasone, and 10 mM β glycerophosphate (all from Sigma-Aldrich) to the respective growth media. Cells in standard growth medium served as controls. After 21 days, extracellular calcium deposition was evaluated via Alizarin red S staining (Sigma-Aldrich). For quantification, the stain was dissolved in cetylpyridinium chloride (Sigma-Aldrich) and absorbance was measured at 540 nm using the microplate reader.

#### Gene expression

After 7 days of osteogenic induction, the expression of osteogenesis-related genes (Supplementary Table 1) was assessed in BMSCs and ASCs in HPL and FBS via quantitative real-time polymerase chain reaction (qPCR) using TaqMan® real-time PCR assays (Thermo Fisher Scientific). RNA extraction and cDNA synthesis were performed as previously described [17]. The expressions of the genes of interest were normalized to that of glyceraldehyde 3-phosphate dehydrogenase (GAPDH). Data were analyzed by the *ΔΔCt* method, and results are presented as fold changes in HPL groups relative to FBS groups.

#### Alkaline phosphatase (ALP) activity

After 7 and 14 days, ALP activity in the cells was measured using the SIGMAFAST BCIP/NBT assay (Sigma-Aldrich). Following manufacturer’s instructions, cells were lysed in 0.1% Triton-X100 buffer, mixed with a working solution containing a phosphatase substrate and alkaline buffer solution, and incubated at 37 °C for 15 min, and absorbance was measured at 405 nm using a microplate reader.

### Cytokine content in HPL

#### Multiplex assay and cytokine network analysis

The concentrations of 48 cytokines (Supplementary Table 2) in HPL were measured using a multiplex immunoassay—Bio-Plex® Pro 48-plex Human Cytokine Screening Panel (Bio-Rad Laboratories, CA, USA) and a Bio-Plex® 200 System (Bio-Rad), according to the manufacturer’s instructions. The cytokines included various GFs, inflammatory mediators, and chemokines involved in regulating MSC growth and function. To validate the multiplex data, concentrations of three selected GFs, namely platelet-derived growth factor BB (PDGF-BB), transforming growth factor-β1 (TGF-β1), and vascular endothelial growth factor (VEGF), were measured in representative batches of HPL via enzyme-linked immunosorbent assay (ELISA) kits (R&D Diagnostics, Wiesbaden, Germany) following the manufacturer’s protocols. Interactions between cytokines were analyzed using the Search Tool for the Retrieval of Interacting Genes/Proteins (STRING) database and online software [19]. Cytokines were clustered according to the Markov Cluster algorithm and the STRING global score as previously reported [20].

#### Screening of different storage times to identify a threshold

The first multiplex assay included several HPL batches produced from PCs with different storage times (range 1–9 months). These HPLs, and corresponding PC units, were identified and screened retrospectively from a biobank, i.e., not collected and intentionally frozen for specific periods of time (as performed later in the study). In order to determine whether the duration of frozen storage of PCs affects the cytokine content of subsequently produced HPL, the storage times were divided into two categories: storage ≤ 4 months and > 4 months. Categorization was based on (a) recommendations regarding “quarantine storage” of GMP-grade blood products which state that the product must only be released if the donors have been tested negative for transmissible diseases twice, i.e., at the time of blood donation and re-tested as negative 4 months (or longer) thereafter [13, 21], and (b) current practices at the HPL production site (Haukeland Hospital Bloodbank), which are in line with the above recommendations.

#### Identifying a specific threshold for PC storage time

Since a preliminary threshold of 4 months was identified in the screening assay, a more focused custom-designed multiplex assay with 16 selected cytokines was performed to identify a specific threshold, if any, between 0 and 4 months. For this purpose, HPL batches were specially produced from PCs frozen for controlled durations of 1, 2, 3, and 4 months. A reference HPL batch of PCs frozen and processed immediately (“0 months”) was also included. The custom assay was a modification of the 48-plex panel (Bio-Rad) previously described. For both multiplex assays, data was analyzed using the Bio-Plex Manager Software (Bio-Rad) and final cytokine concentrations were derived in pg/mL.

### Effect of frozen PC storage time on HPL efficacy

#### MSC morphology and proliferation kinetics

To investigate whether PC storage times affected the biological performance of HPL, cellular assays were performed using BMSCs. Previously cryopreserved passage 1 BMSCs were expanded for three additional passages in HPL produced from 0-, 1-, 2-, 3-, or 4-month PCs. At approximately 80% sub-confluence, cells from all conditions were harvested, counted, and re-seeded at 4000 cells/cm^2^, following the same clinically validated protocol [11]. The population doubling (PD) rate was determined using the following formula [22]:

$$ X=\frac{\log 10\left({N}_H\right)-\log 10\left({N}_1\right)}{\log 10(2)} $$

*N*_*H*_ is the harvested cell number and *N*_*1*_ is the plated cell number. The PD for each passage was calculated and added to the PD of the previous passages to generate data for cumulative population doublings (CPD). Additionally, the population doubling time (PDT), i.e., the average time between two doublings, was calculated using the following formula [22]:

$$ X=\frac{\log 2\times \varDelta t}{\log 10\left({N}_H\right)-\log 10\left({N}_1\right)} $$

#### MSC osteogenic differentiation

To investigate whether PC freezing times affected the osteogenic differentiation potential of BMSCs, cells expanded for two passages with HPL produced from 0-, 1-, 2-, 3-, or 4-month PCs were plated for osteogenic differentiation assays. The differentiation medium was prepared by adding osteogenic supplements (as described above) to the respective growth media. Osteogenic differentiation was assessed via an ALP assay after 7 and 14 days (as described above) and via Alizarin red S staining of extracellular calcium deposits after 21 days (as described above) in osteogenically induced and non-induced BMSCs. Additionally, quantification of DNA per sample in the ALP experiment was performed as previously described. ALP activity was normalized to the amount of DNA per corresponding sample (ng/mL).

### Statistical analysis

Statistical analyses were performed using the IBM SPSS version 17.0 software package (SPSS Inc., Chicago, IL, USA). Data are represented as arithmetic means ± SD, unless specified. For gene expression, statistical analyses are based on delta-Ct values and data are presented as relative fold changes. The student *t* test and one-way analysis of variance (ANOVA), followed by a post hoc Tukey’s test for multiple comparisons, were applied when appropriate and *p* < 0.05 was considered statistically significant.

## Results

### Characterization of HPL efficacy

#### Isolation and characterization of BMSCs and ASCs

**Fig. 1:**
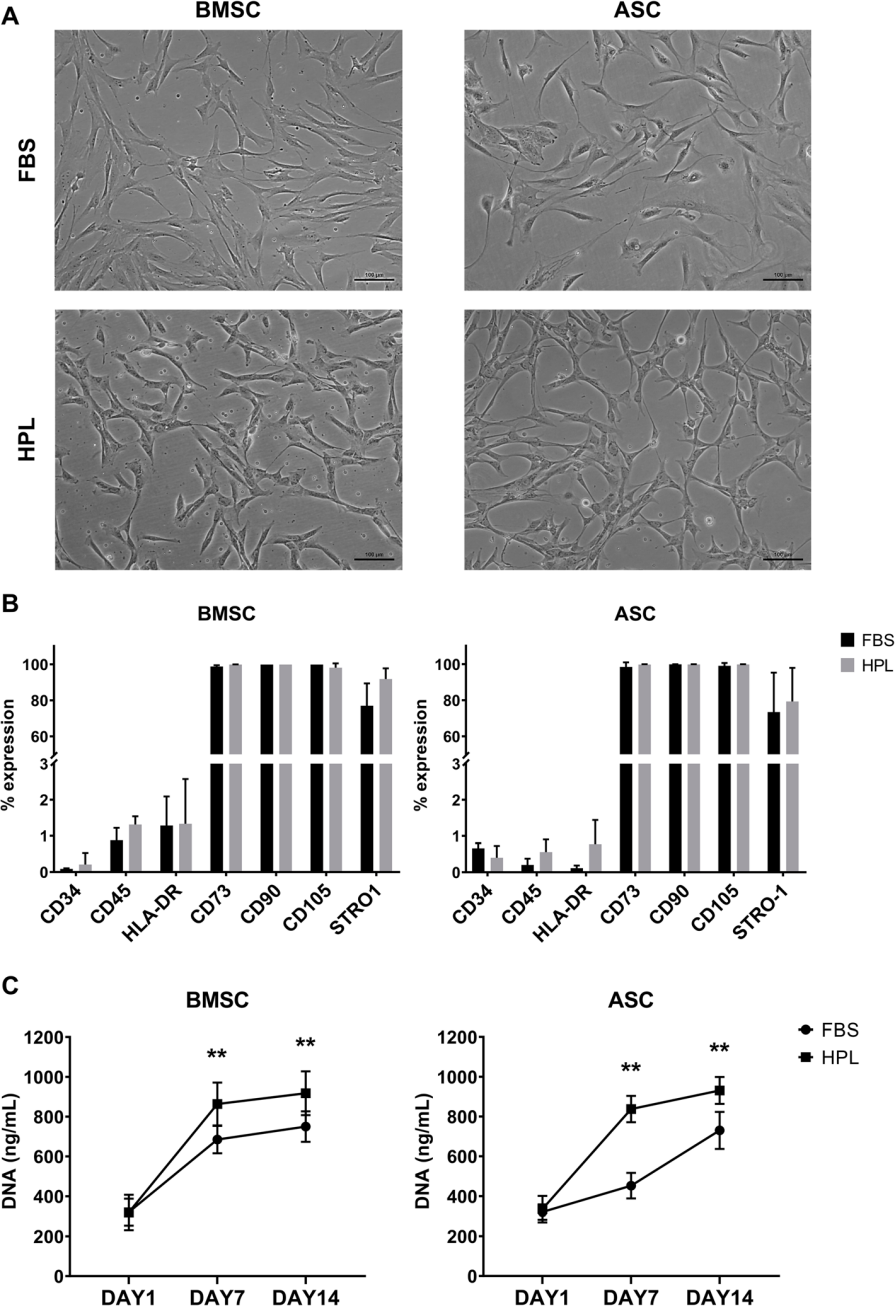
Characterization of BMSCs and ASCs in HPL. **a** Morphology of BMSCs and ASCs from one representative donor (scale bar 100 μm). **b** Surface marker expression of BMSCs and ASCs based on flow cytometry; data represent means ± SD (*n* = 3 donors). **c** Proliferation of BMSCs and ASCs over 14 days based on DNA quantification; data represent means ± SD (*n* = 3 donors); **p < 0.001

Donor-matched BMSCs and ASCs demonstrating characteristic plastic adherence and fibroblastic morphology were successfully expanded in both HPL- and FBS-supplemented media. Distinct morphological differences were observed between cells in HPL and FBS—the former being smaller and more spindle-shaped; these differences were more apparent at earlier passages (Fig. 1a). BMSCs and ASCs in both HPL and FBS demonstrated the characteristic MSC phenotype, i.e., > 95% of the cells were positive for the stromal markers CD73, CD90, and CD105, while < 5% of the cells expressed HLA-DR or the hematopoietic markers CD34 and CD45 (Fig. 1b, Supplementary figure 1). A trend for higher expression of STRO-1 was observed in HPL-cultured BMSCs and ASCs (Fig. 1c). Cell proliferation over 14 days was significantly greater in HPL-cultured BMSCs and ASCs based on DNA quantification (Fig. 1c).

#### Multi-lineage differentiation of BMSCs and ASCs

**Fig. 2:**
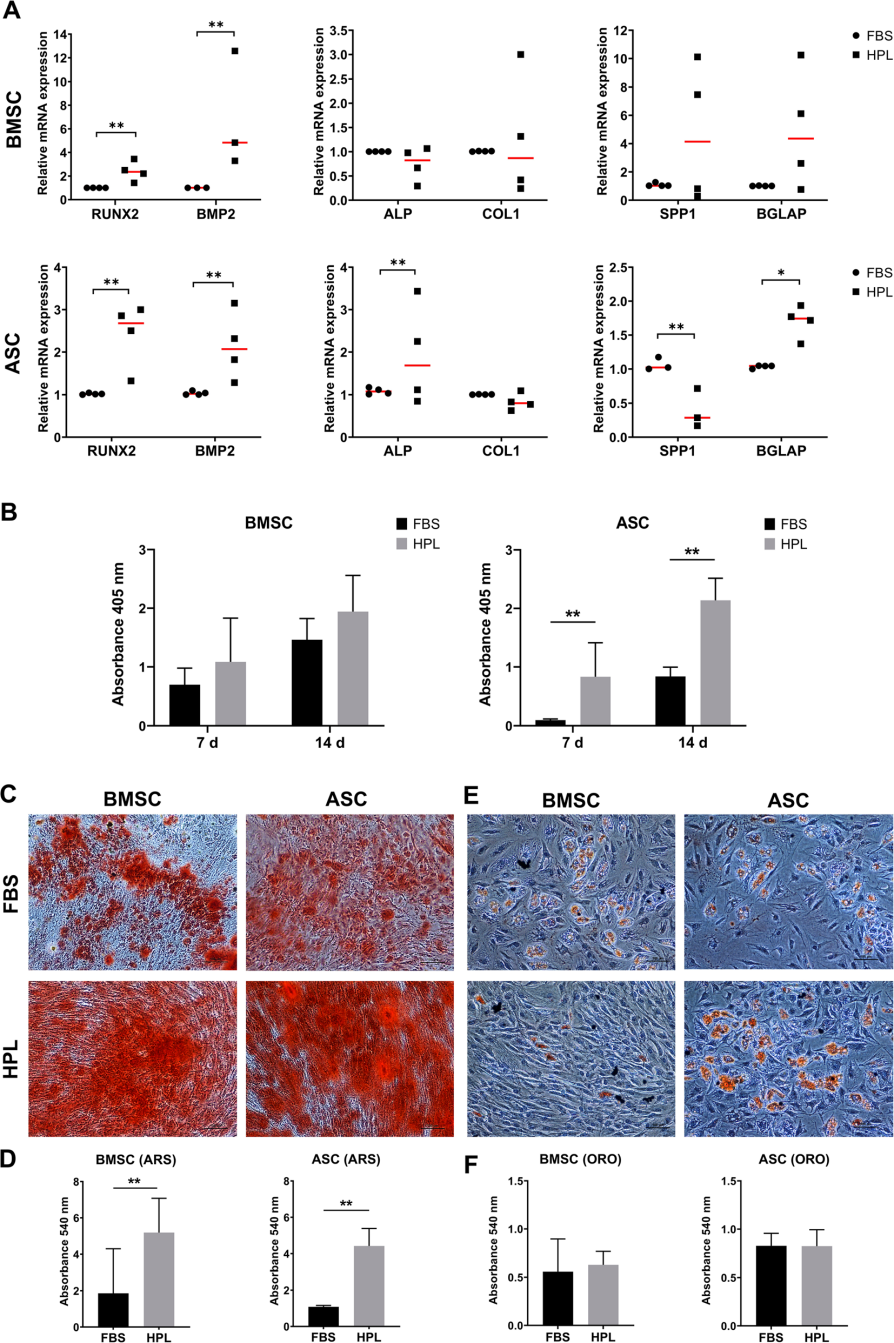
Multi-lineage differentiation of BMSCs and ASCs in HPL. **a** Osteogenic differentiation: relative expression (fold changes) of early, intermediate, and late osteogenic gene markers in BMSCs and ASCs after 7 days of induction. Data represent means; each symbol represents a single donor (*n* = ≥ 3 donors) based on the average of ≥ 2 experimental replicates; statistical analyses are based on delta-Ct values; *p < 0.05; **p < 0.001. **b** ALP activity in BMSCs and ASCs after 7 and 14 days of osteogenic induction. Data represent means ± SD (*n* = 3 donors); **p < 0.001. Representative images of Alizarin Red S (ARS) staining (**c**) and quantification (**d**) after 21 days. **e** Adipogenic differentiation: representative images of Oil red O (ORO) staining and quantification (**f**) after 14 days. Scale bars 100 μm. Data represent means ± SD (*n* = 3 donors); **p < 0.001

**Fig. 3:**
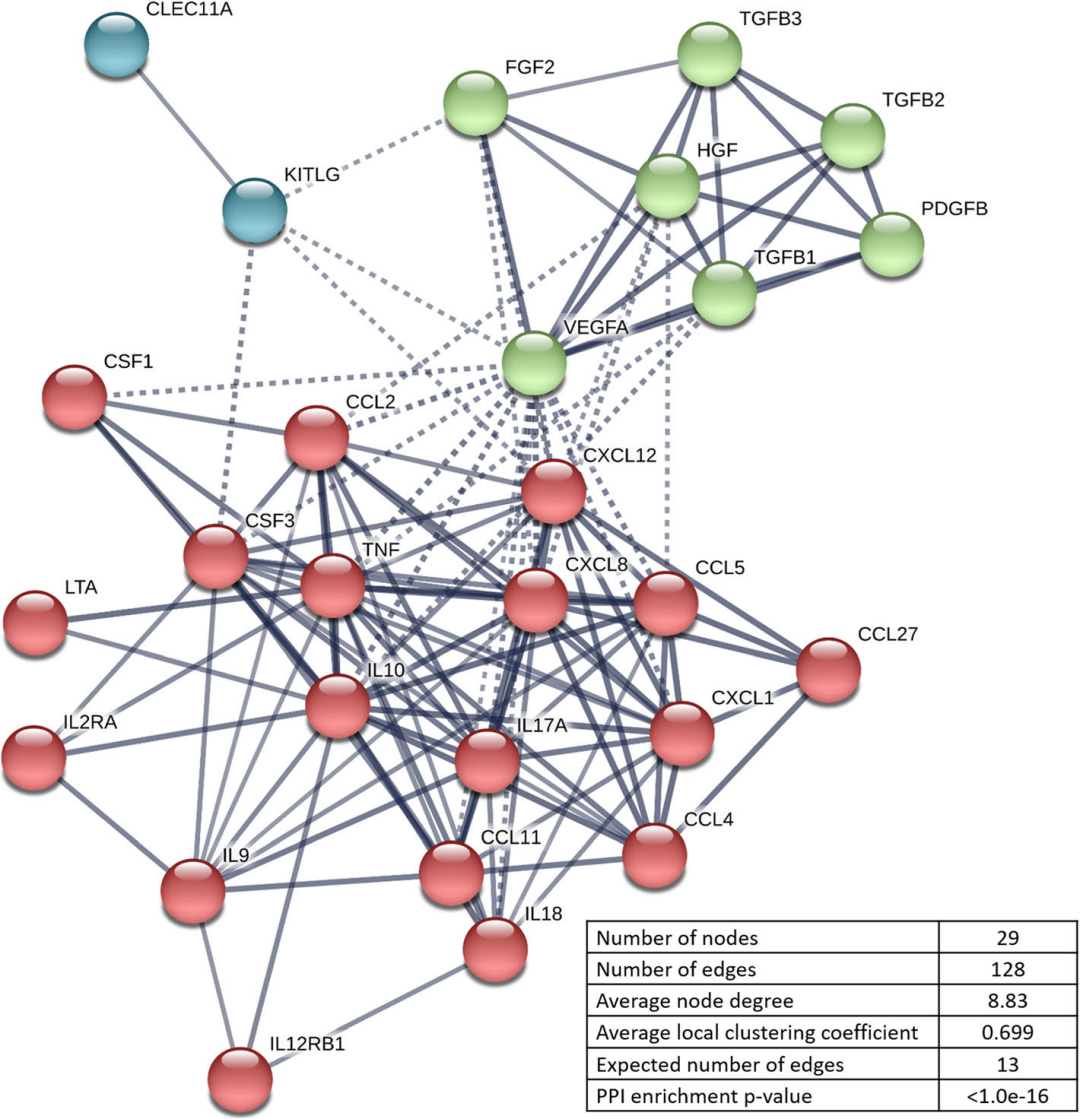
Protein–protein interaction network visualized by STRING. The nodes indicate proteins, and edges indicate the number of interactions. Color saturation of the edges represents the confidence score of a functional association. The number of average interactions per node is indicated by the node degree. The clustering coefficient specifies the average node density of the map. Disconnected nodes are hidden, and only interactions with a high confidence score of ≥ 0.7 are shown

BMSCs and ASCs in both HPL- and FBS-supplemented media demonstrated the capacity to differentiate into adipocytes and osteoblasts, with some differences. Osteogenic differentiation in HPL and FBS was assessed at the gene, protein, and functional levels. Expression of early osteogenesis-related genes RUNX2 and BMP2 was significantly upregulated in HPL-cultured BMSCs and ASCs after 7 days (Fig. 3a). Interestingly, expressions of SPP1 and BGLAP, typically associated with later stages of osteogenesis, were also upregulated in HPL-cultured cells; BGLAP was significantly upregulated in ASCs. Intracellular ALP activity after 7 and 14 days was higher in HPL- vs. FBS-cultured BMSCs and ASCs; these differences were more pronounced in ASCs (Fig. 3b). While BMSCs generally presented higher ALP activity compared to ASCs at 7 days, the activity at 14 days was comparable between the two cell types. Significantly greater mineral deposition via Alizarin red S staining was observed in HPL- vs. FBS-cultured BMSCs and ASCs after 21 days, suggesting an enhanced osteogenic differentiation capacity of these cells (Fig. 3c). A trend for superior mineralization was observed in BMSCs as compared to ASCs. After 14 days of induction, ASCs demonstrated superior adipogenic differentiation, i.e., greater accumulation of intracellular lipid vesicles, compared to BMSCs, as revealed by quantification of Oil red O staining (Fig. 2e). HPL-cultured ASCs and BMSCs demonstrated similar adipogenic differentiation vs. their FBS-cultured counterparts (Fig. 2f). No adipogenic or osteogenic differentiation of cells was observed in the standard growth media (data not shown).

### Characterization of HPL cytokine content

#### Multiplex assay and cytokine network analysis

A multiplex immunoassay was performed using HPLs produced from frozen PCs stored for 1–9 months. Thirty of the 48 cytokines tested, including various GFs (*n* = 11), chemokines (*n* = 9), and inflammatory mediators (*n* = 10), were reliably detected in all tested HPLs. Cytokine concentrations, in comparison to previous studies, are reported in Table 1. Concentrations of three selected GFs, i.e., PDGF-BB, TGF-1, and VEGF, were validated via ELISA (Supplementary figure 2). The cytokine network analysis identified two major clusters of GFs, and chemokine/inflammatory mediators; stem cell growth factor (SCGF/CLEC11A) and stem cell factor (SCF/KITLG) were clustered separately (Fig. 3). Clear and abundant interactions were identified between the clusters including synergistic relations between several proteins that contribute to MSC proliferation, chemotaxis, and osteogenic differentiation.

**Table 1:** Multiplex-based measurements of cytokine concentrations (pg/mL) in HPL

Reference	(21)*	(39)	(41)	(42)	(43)	(44)**	Present study
Starting material	< 5 d BC or AP	< 24 h AP	Fresh BC	Exp BC	7 d BC (3 w at − 80C), pathogen inactivated	5–7 d BC	7 d BC
Donors (*n)*	< 12 (BC) or 1 (AP)	1	16	245 + 16	16	40	20
Lysis method	1–2× F/T	2× F/T	3× F/T	1× F/T	3× F/T	3× F/T	3× F/T
Cytokines (*n*)	23	12	27	22	37	45	48
PDGF-AA	239,412 + 53,690			10,287 + 1820	11,433.75 + 3083.45		
PDGF-AB/BB	571,730 + 381,036	1244 + 478.46	13,534.4 + 326.9	27,407 + 5365	25,941.5 + 1891.06	11,121 + 1126	11,783.482 + 917.39
TGF-β1	139,029 + 18,854						306,801.77 + 81,171.87
b-FGF	495 + 27	77.09 + 21.33	256.6 + 7.6		407 + 105	569 + 10	56.48 + 9.85
HGF			1594.7 + 172.3			2631 + 204	542.39 + 42.21
VEGF-A/D	325 + 34	660.88 + 221.90	421.9 + 1.9		424.5 + 88.91	1742 + 133/398 + 60	440.175 + 40.35
EGF			754.9 + 89.9		997.5 + 825.58	1104 + 224	
IGF						1122 + 54	
b-NGF		85.55 + 24.27				936 + 28	19.05 + 9.29
BDNF						3169 + 213	
SCGF/CLEC11a							186,005.65 + 12,463.91
SCF/KITLG						260 + 35	30.45 + 4.35
G-CSF	74 + 19		131.4 + 9.4		40 + 15.36		108.68 + 13.17
GM-CSF	34 + 16		98.1 + 3.8		22 + 6.27	2423 + 0	7.42 + 2.28
M-CSF				129,689 + 14,654			129.65 + 55.04
MCP1/CCL2		585.75 + 200.47	64.5 + 5.0		152.5 + 30.65	1060 + 73	16.00 + 3.26
MIP-1α/CCL3	47 + 4		12.5 + 0.5	29,337 + 2030	27.25 + 5.12	531 + 37	1.59 + 0.24
MIP-1β/CCL4	51 + 5		134.9 + 2.3	17,087 + 2385	124.25 + 33.93	1641 + 289	169.77 + 13.01
RANTES/CCL5	2,705,600 + 496,076	67.71 + 18.33	15,810.8 + 717.7	376,730 + 56,734		1453 + 24	8788.00 + 644.50
MCP3/CCL7					397 + 126.25		OOR<
Eotaxin/CCL11			72.6 + 3.3		91.5 + 31.2	196 + 64	44.68 + 5.86
CTACK/CCL27							311.83 + 44.73
MSP/MST1				688,589 + 132,037			
MDC					470.25 + 300.42		
MIF				287,188 + 51,282			6645.36 + 768.15
LIF						1473 + 114	79.47 + 18.88
GROa/CXCL1	11,126 + 6480			40,947 + 3148		866 + 109	1203.04 + 98.03
IL-8/CXCL8	80 + 6	17.15 + 5.22	112.5 + 5.3		57 + 16.53	ND	21.98 + 3.82
MIG/CXCL9							96.33 + 8.36
IP-10/CXCL10			284.7 + 3.1		82.5 + 33.37	527 + 65	384.76 + 11.42
SDF1α/CXCL12						16,102 + 1506	753.49 + 49.21
Fractalkine/CX3CL1					174.75 + 54.59		
IL-1α	41 + 6	88.78 + 33.30		4854 + 533	39.25 + 18.44	ND	
IL-1β	3 + 2	24.89 + 9.22	6.7 + 0.4		4.47 + 1.77	ND	2.82 + 0.39
IL-1ra			235.3 + 4.8	3997 + 589	717.25 + 283.94	10,580 + 605	
IL-2	OOR<		OOR<		4.92 + 2.59	ND	OOR<
IL-2ra							209.18 + 81.59
IL-3					4.97 + 1.55		OOR<
IL-4			14.2 + 0.5	3840 + 639	30.75 + 12.91	ND	OOR<
IL-5			OOR <		53.25 + 26.34	ND	180.33 + 67.29
IL-6	3 + 0	159.75 + 61.57	22.5 + 0.6		9 + 4.42	1847 + 178	54.19 + 21.40
IL-7	32 + 16		41.8 + 1.1		27 + 7.39	145 + 24	31.43 + 5.57
IL-9			129.9 + 6.3		6.9 + 2.5	942 + 49	208.42 + 20.72
IL-10	3 + 2		60.2 + 2.4		10.85 + 7.74	186 + 25	OOR <
IL-12(p40)					51.5 + 13.91		135.82 + 25.12
IL-12(p70)			113.9 + 5.1		8.85 + 3.08	ND	12.85 + 4.61
IL-13			7.7 + 1.1		291 + 131.16	ND	OOR<
IL-15			OOR <		7.7 + 3.52	568 + 29	689.27 + 228.99
IL-17			1022.5 + 56.4		10.87 + 4.04	622 + 91	11.25 + 1.76
IL-18						2466 + 349	34.37 + 11.56
IL-21						ND	
IL-22						ND	
IL-23						ND	
IL-27						2658 + 1053	
IL-31						ND	
TNF-α	8 + 2	427.25 + 167.01	133.3 + 10.4		20.25 + 5.56	2942 + 0	46.97 + 5.8
TNF-β					390.5 + 164.81	ND	246.03 + 25.05
TRAIL/TNFSF10							86.28 + 5.33
IFN-γ	14 + 4	6.61 + 2.27	154.6 + 7.4		12.125 + 2.59	ND	23.41 + 3.19
IFN-a2					63.25 + 19.72	64 + 40	8.44 + 1.29
VCAM-1	1,789,695 + 1,108,320						
ICAM-1	137,300 + 93,670						
Angiopoietin-1				121,156 + 22,164			
Angiogenin				102,085 + 17,627			
IGFBP3				530,240 + 75,663			
CD40L	29,738 + 8361			151,662 + 17,153			
TIMP-1				231,407 + 39,966			

*BC* buffy coats, *AP* apheresis, *PI* pathogen inactivated, *F/T* freeze/thaw cycles, *d* days, *w* weeks, *OOR* out of range

Data represent means ± SD

*No significant differences between buffy coat- and apheresis-derived HPL

**Cytokine concentrations in medium supplemented with 10% HPL

#### Screening of different storage times to identify a threshold

**Fig. 4:**
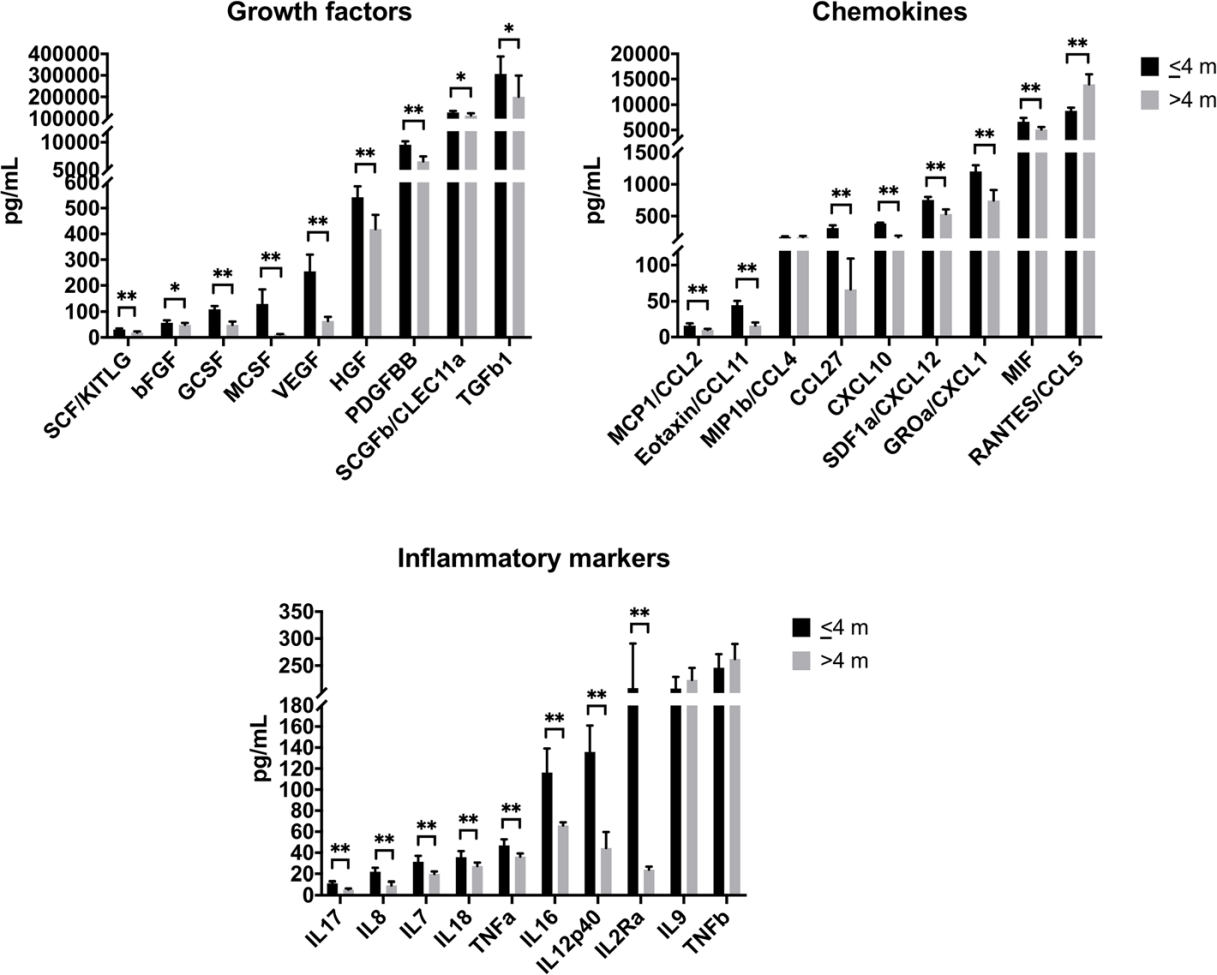
Cytokine concentrations in HPL from PCs stored for ≤ 4 or > 4 [5–9] months (m). Data represent means ± SD (*n* = 12 HPL batches per group). *p < 0.05, **p < 0.001

Of these 30 cytokines, the concentrations of 27 cytokines were significantly reduced in the > 4-month group while only one cytokine, i.e., regulated upon activation, normal T cell expressed and secreted (RANTES), was significantly increased vs. the ≤ 4-month group. In addition to the known predominant cytokines PDGF-BB and TGF-β1, high levels of SCGF and macrophage inhibitory factor (MIF) were detected in HPL. Other GFs, such as basic fibroblast growth factor (b-FGF), hepatocyte growth factor (HGF), SCF, VEGF, and all inflammatory mediators [various interleukins (IL), tumor necrosis factor-α (TNF-α), and TNF-β] were present in relatively lower concentrations (Fig. 4).

#### Identifying a specific threshold for PC storage time

**Fig. 5:**
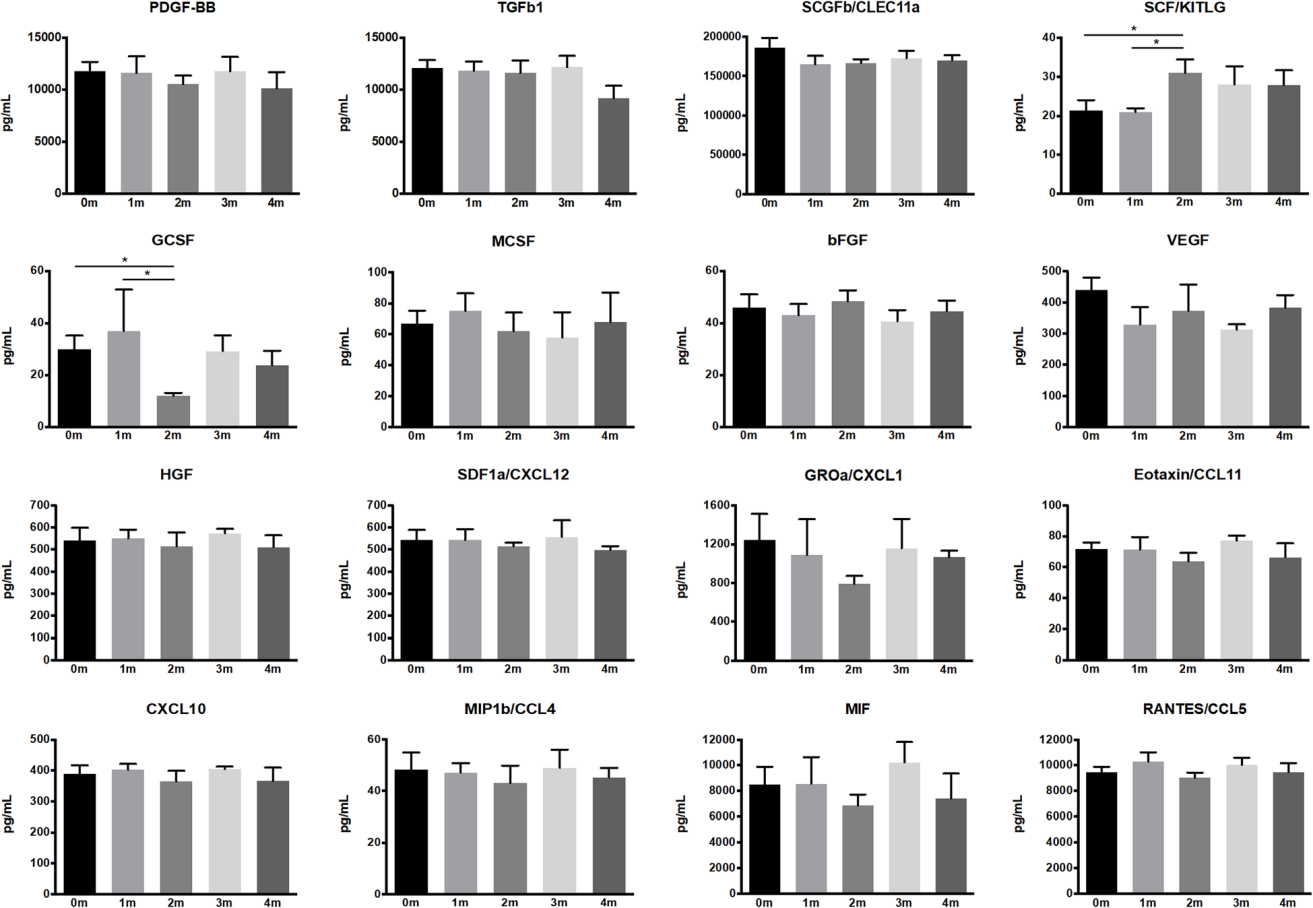
Cytokine concentrations in HPL from PCs stored for 0–4 months (m). Data represent means ± SD (*n* = ≥3 HPL batches per group). *p < 0.05

After a preliminary threshold of 4 months was identified, a second multiplex immunoassay with 16 selected cytokines was performed to identify a specific threshold, if any, for cytokine degradation between 0 and 4 months. Significantly lower concentrations were detected at 0 and 1 months for SCF and at 2 months for GCSF (Fig. 5). No significant differences were observed between the different storage times for any of the other tested cytokines, and no definitive threshold below 4 months could be identified.

### Effect of frozen PC storage time on HPL efficacy

#### MSC morphology and proliferation kinetics

**Fig. 6:**
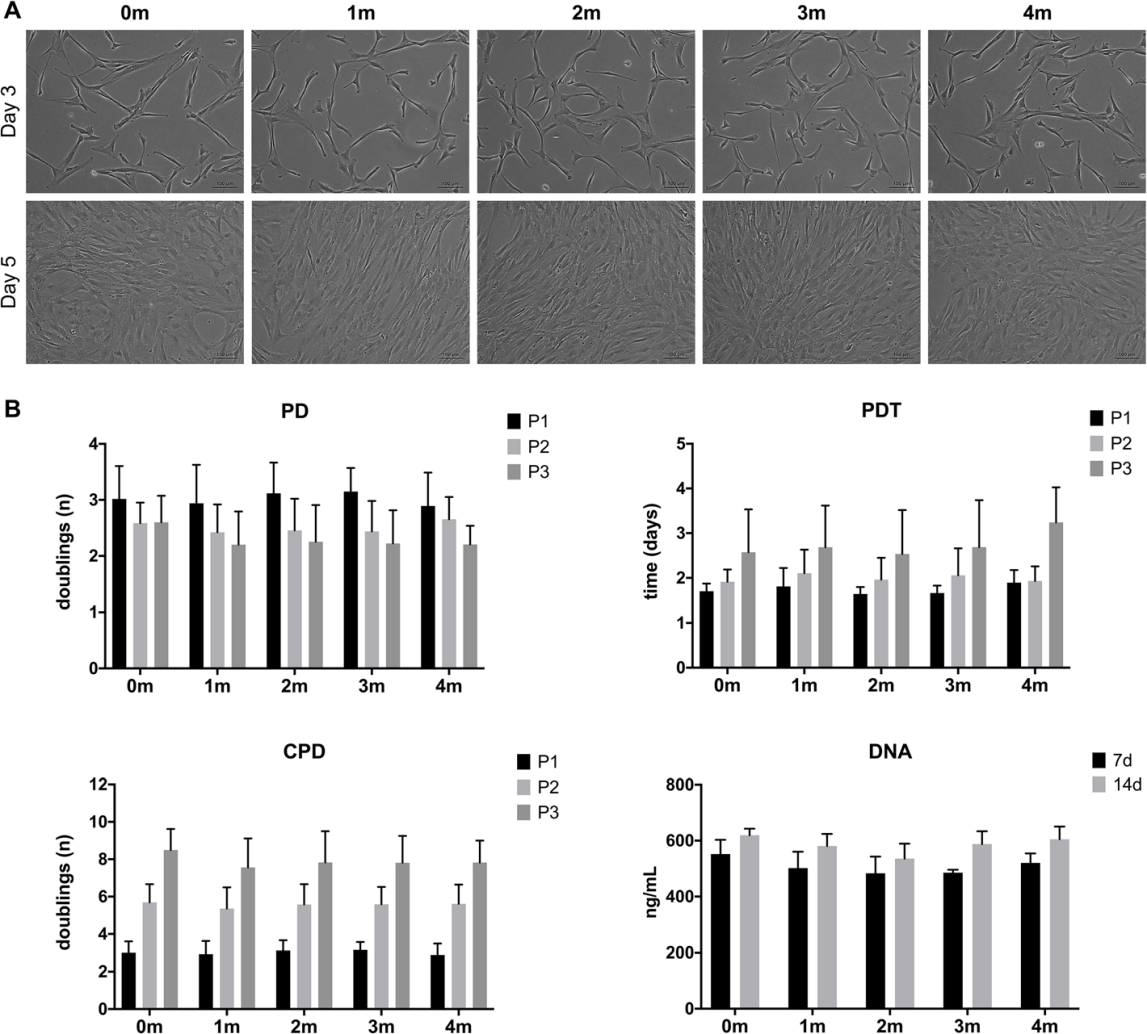
Morphology and proliferation kinetics of BMSCs in HPL from PCs stored for 0–4 months (m). **a** Morphology of BMSCs at passage 2 (representative images from one donor), scale bar 100 μm. **b** Proliferation kinetics over three serial passages (P1–3) and DNA content after 7 and 14 days (d). Data represent means ± SD (*n* = ≥ 4 donors). PD, population doubling rate; CPD, cumulative population doublings; PDT, population doubling time

PC storage time did not seem to affect the biological performance of HPL; no differences in BMSC morphology were observed between the different storage times over three serial passages (Fig. 6a). The proliferation data revealed lower PD rate (fewer doublings) and higher PDT with increasing passages. No significant differences were observed with regard to kinetics-related variables (PD, CPD, PDT) or absolute DNA amounts between the different PC storage times (Fig. 6b).

#### MSC osteogenic differentiation

**Fig. 7:**
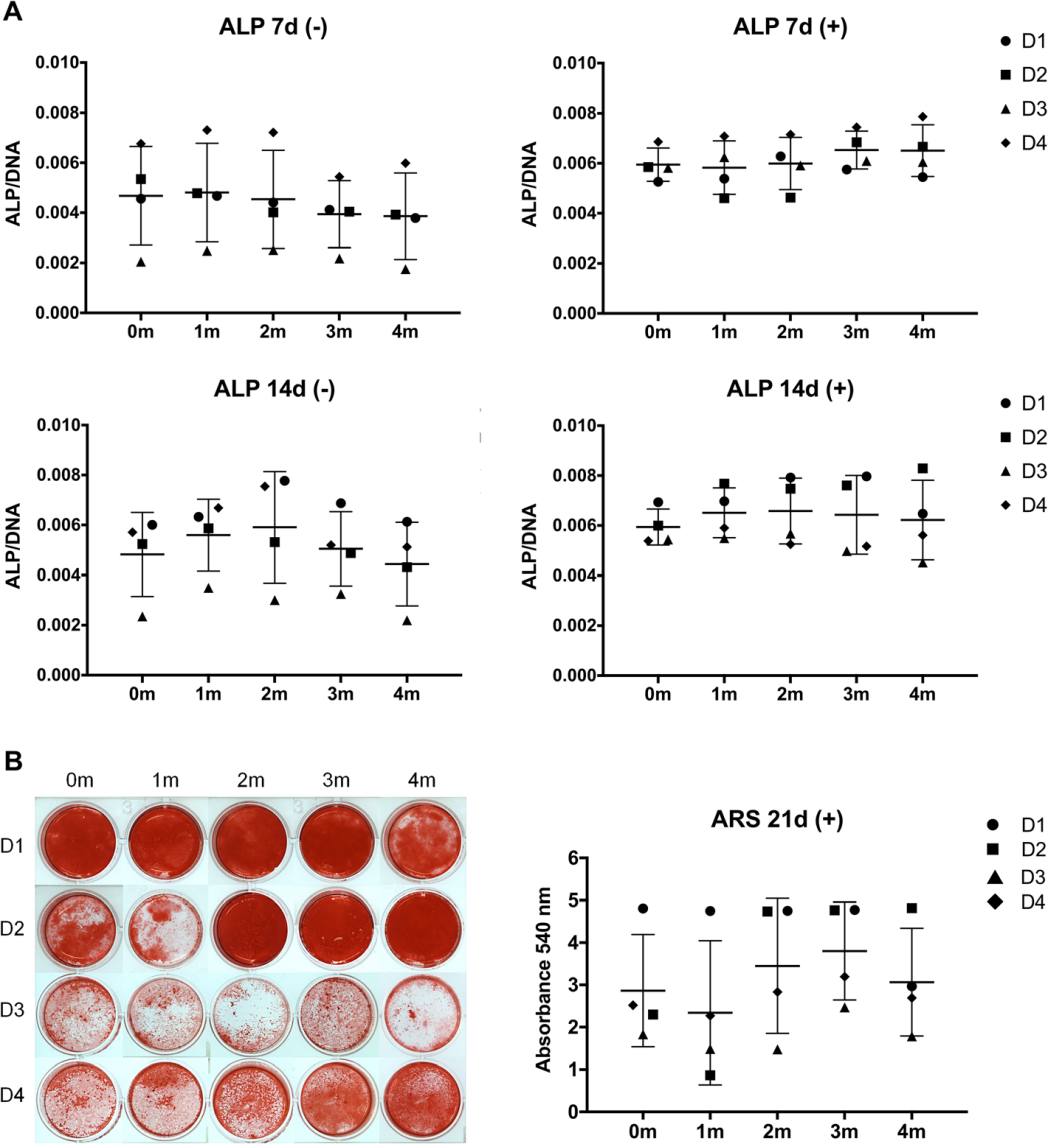
Osteogenic differentiation of BMSCs in HPL from PCs stored for 0–4 months (m). **a** ALP activity at 7 and 14 days (d) in osteogenic (+) and standard (−) HPL media. ALP activity (absorbance) was normalized to the corresponding DNA content (ng/mL). **b** Representative images and quantification of Alizarin Red S (ARS)-stained cells after 21 days of induction. Data represent means ± SD; each symbol represents a single donor (D, *n* = 4 donors) based on the average of 3 experimental replicates

To investigate whether PC storage times affected the osteogenic differentiation potential of BMSCs, ALP activity (7, 14 days) and mineralization (21 days) were assessed. When combining data from all donors, no significant differences in ALP (Fig. 7a) or mineralization (Fig. 7b) were observed between the different PC storage times. Considerable variation was observed between the different BMSC donors in all groups—a trend for higher mean ALP activity (at 7 days) and mineralization, with lower inter-donor variation, was observed in the 3-month storage group. When analyzing data from individual donors, some differences in ALP activity and mineralization were observed, i.e., BMSCs from the same donor showed different activities in HPLs from different PC storage times, although these differences did not reach statistical significance for any of the donors. Overall, donor-related properties rather than PC storage time seemed to influence the osteogenic potential of HPL-cultured BMSCs.

## Discussion

HPL is emerging as the preferred xeno-free supplement for the GMP-grade expansion of MSCs for BTE applications [1, 11]. Accordingly, there is a growing need for standardization and scaling-up of HPL production [12, 14]. Current GMP guidelines call for HPL release criteria to include testing for specific cytokines and biological efficacy based on cellular assays [9, 12]. In the present study, a scalable and GMP-compliant HPL was produced based on previously published methods and characterized for its cytokine content and efficacy for MSC expansion. Consistent with previous reports, HPL supported the expansion, stromal phenotype, and multi-lineage, particularly osteogenic, differentiation of MSCs in comparison to FBS [17].

A strength of the present study was the comparison of donor-matched cells from two different tissue sources, i.e., BMSCs and ASCs, to evaluate HPL efficacy. Moreover, MSCs from each tissue type were cultured in HPL- and FBS-supplemented media from the time of isolation (passage 0), thus allowing true and standardized comparisons between xeno-free and xenogeneic-cultured cells [23]. Since the focus herein was BTE, the in vitro osteogenic differentiation of BMSCs and ASCs was studied in detail and was shown to be significantly enhanced in HPL vs. FBS at the early (expression of osteogenic genes), intermediate (ALP activity) and late stages (mineral deposition). Moreover, a trend for higher expression of STRO-1, a marker associated increased osteogenic potential [24], was observed in HPL- vs. FBS-cultured BMSCs and ASCs. When comparing the two cell types, osteogenic differentiation appeared to be accelerated in HPL-cultured BMSCs vs. ASCs, based on gene expression and ALP activity during the “early” differentiation stages, while adipogenic differentiation of HPL-cultured ASCs was superior to that of BMSCs. One possible explanation could be the “tissue source variability” of BMSCs and ASCs [17, 25]. In context, previous studies have reported similar or enhanced differentiation of ASCs compared to BMSCs in vitro, but inferior bone formation in vivo, in both xenogeneic [26, 27] and HPL-supplemented cultures [28].

A substantial body of evidence points to the enhanced osteogenic potential of MSCs cultured in HPL [29–36], although the specific components contributing to this phenomenon are unknown. In the present study, the cytokine content of HPL was analyzed via a quantitative multiplex immunoassay to identify potentially relevant cytokines contributing to MSC osteogenesis. Although previous studies have measured cytokines in HPL via semi-quantitative assays [22, 36–40], to our knowledge, only five studies have reported quantitative multiplex-based assessments [21, 39, 41–44]. Considerable differences in cytokine concentrations are observed across the different studies (Table 1). Moreover, it is presently unclear which cytokines in HPL are most important, what are the optimal (minimum and/or maximum) concentrations of specific cytokines, and what are the effects of HPL preparation methods on individual cytokine concentrations [10]. Nevertheless, some cytokines such as PDGF-BB, TGF-β1, and b-FGF have been consistently identified in HPL in substantial quantities. A previous study identified PDGF-BB, TGF-β1, and b-FGF to be necessary for the optimal proliferation of MSCs in HPL [21]. However, these three factors on their own were not sufficient to promote MSC proliferation [21]. These data are consistent with findings that combinations of cytokines, rather than single GFs, are important to exert maximal effects on MSC migration and proliferation [45]. However, in another study, even the use of defined combinations of several recombinant GFs and chemokines was inferior to HPL supplementation for MSC expansion [46]. Since measurement of selected cytokine concentrations has been cited as a “quality control” measure for GMP-grade HPL [12], further information is needed on which cytokines (for specific MSC applications, e.g., BTE) should be tested along with “target” concentration ranges.

In addition to established factors such as PDGF-BB and TGF-β1, high concentrations of stem cell growth factor (SCGF)—a cytokine not previously identified in HPL—were detected in the multiplex analysis herein. SCGF is a protein encoded by the CLEC11A gene (C-type lectin domain family 11, member A) and is associated with the growth of hematopoietic progenitor cells [47]. In the context of the bone, SCGF/CLEC11A is reportedly expressed in the bone marrow by a variety of stromal cells [47, 48]. Interestingly, CLEC11A was recently shown to be expressed by murine BMSCs, and its overexpression promoted their in vitro osteogenic differentiation and in vivo osteogenesis in a fracture healing model [47]. However, a more recent study showed contrasting results in human BMSCs, where silencing, rather than overexpression, of CLEC11A promoted their in vitro osteogenic differentiation [49]. In another study, SCGF was detected in the secretome of BMSCs undergoing osteogenic differentiation and was found to be downregulated on days 1, 7, and 14 compared to day 0 [50]. Thus, in addition to PDGF-BB and TGF-β1, SCGF/CLEC11A signaling may be involved in the regulation of osteogenic differentiation of HPL-cultured MSCs.

Consistent with results from the above study [49], the in silico network analysis herein identified only a single interaction for SCGF/CLEC11A, which was with the chemokine stem cell factor (SCF), a ligand for the c-kit receptor (KITLG) [51]. Like SCGF, SCF is also typically associated with hematopoietic cell proliferation [51]. Although SCF was detected at a relatively lower concentration compared to SCGF, the network analysis revealed several interactions with the cytokine/chemokine and GF clusters. Recently, SCF signaling has been implicated in the mobilization, and subsequent osteogenic differentiation, of BMSCs in vitro and in in vivo models of fracture healing [52] and dental pulp/dentin regeneration [53]. Further studies are needed to elucidate the nature of the interaction(s) between SCGF, SCF, and other cytokines in the context of MSCs’ osteogenic differentiation.

In addition to GFs, HPL also contains a wide range of chemokines, which regulate MSC migration, proliferation, and differentiation. Several chemokines of the CCL and CXCL families have been identified in HPL (Table 1). Of these, stromal derived factor-1 (SDF-1/CXCL12) is the most extensively studied and is involved in the recruitment of endogenous BMSCs to injury sites [54]. Platelets have been shown to release SDF1 and recruit progenitor cells to initiate wound healing at sites of vascular injury [55]. In the context of the bone, SDF1 was shown to play a critical role in the recruitment of murine BMSCs to the injury site during the early stages of fracture healing, and inhibition of SDF1 led to reduced in vivo bone formation [56]. Moreover, SDF1 regulated BMP2-induced osteogenic differentiation of mouse and human BMSCs; blocking SDF1 signaling led to significantly reduced ALP activity and mineralization of the cells [57]. Recent studies have also demonstrated enhanced in vivo bone regeneration following delivery of SDF1 via recruitment of endogenous MSCs to regeneration sites [58–61], thus highlighting the role of SDF1 in regulating MSCs’ osteogenic differentiation.

Emerging evidence suggests that MSCs exert their regenerative effects primarily via paracrine mechanisms and modulation of immune cells, including osteoclasts [62]. Osteoblast-osteoclast interactions are known to be critical for bone regeneration. This is especially relevant in BTE, where MSCs are often delivered using biomaterial scaffolds, which elicits an initial inflammatory/resorptive response by macrophages/osteoclasts prior to bone formation by MSCs/osteoblasts [63]. It is therefore also of interest to consider the cytokines in HPL that may be involved in the regulation of osteoclastic activity. The most consistently reported of these are RANTES/CCL5 and associated cytokines, monocyte chemotactic protein-1 (MCP-1/CCL2), macrophage inflammatory protein 1 (MIP-1α/CCL3 and MIP-β/CCL4), and macrophage migration inhibitory factor (MIF). All of these have been implicated in the recruitment and differentiation of osteoclasts and/or their precursors [64–66]. Moreover, it has been demonstrated that RANTES secreted by osteoclasts promotes the migration of osteoblasts and MSCs in vitro [64, 67, 68] and mineralization in vivo [68, 69].

In addition to GFs and chemokines, a number of inflammatory cytokines were identified in the HPL herein. The evidence for the effects of inflammatory cytokines on MSCs is conflicting since these effects appear to be (a) tissue/site-specific, (b) MSC type-specific, and (c) dose-dependent, based on which a particular cytokine may exert pro- or anti-inflammatory and pro- or anti-osteogenic effects [54]. The most commonly reported of these are TNF-α and IL-1, predominant during the acute inflammatory phase of healing. The combination of HPL and exogenous IL-1α was shown to result in a transient increase in the inflammatory response accompanied by an increase in proliferation, without loss of differentiation potential, in human osteoblasts [70] and ASCs [71]. Interferon-γ (IFN-γ), another major pro-inflammatory cytokine, has consistently more anti-osteogenic effects [54]. Nevertheless, several studies have reported advantages of “pre-conditioning” MSCs with IFN-γ, either alone or in combination with other cytokines such as TNF-α and IL-1, in terms of their immunomodulatory and regenerative potential [72].

Recent studies have reported differences in MSC proliferation and osteogenic differentiation when cultured in different HPL formulations, expressing differences in their protein compositions [15, 73]. MSC proliferation, i.e., PD rate/time, is considered a “key parameter” during ex vivo expansion [11], and ALP and mineralization assays are routinely used to test the in vitro osteogenic capacity of MSCs. In the context of BTE, the in vitro PD time and ALP activity of MSCs are reported to most likely correlate with their in vivo mineralization capacity [74]. Accordingly, in the present study, the growth kinetics and osteogenic potential of BMSCs were tested in HPLs produced from the different PC storage times; BMSCs from multiple donors were used to account for donor-related variations. No significant differences were observed between the different PC storage times in terms of either BMSC proliferation or ALP activity/mineralization. However, considerable donor-related variation was observed in relation to the latter. Notably, the highest relative mean ALP activity and mineralization, with the least inter-donor variation, was observed in the 3-month PC storage group. The results herein are consistent with a recent study reporting on MSCs from a similar donor cohort (healthy young patients), which reported large inter-donor variations in xenogeneic MSCs [17]. It is well-known that several biological (age, sex), behavioral (alcohol/tobacco use), and disease-related (obesity, diabetes) factors influence MSC properties including proliferation and osteogenic differentiation [75]. Nevertheless, it must be acknowledged that the observed donor variation may have confounded the detection of significant differences between PC storage times in the present study.

Among various aspects of HPL production which require standardization is the storage time of the source material, i.e., PCs produced by blood establishments. Current recommendations call for blood centers to freeze outdated PCs (within 7 days of collection) for later HPL production, although “the maximum period time that PCs can be used after expiry to prepare an efficient HPL for cell expansion is unknown” [9]. International blood authorities advise a minimum interval of 3 months between blood donations to allow for repeated viral testing to minimize the risk of disease transmission via platelet products. In the context of HPL, this is especially relevant when smaller PC-pool sizes are used (≤ 16 donors) and where pathogen reduction is not applied [6]. In the present study, HPL produced from PCs stored for > 4 months showed a significant deterioration of several cytokines relevant for MSCs. No significant differences between PC storage times < 4 months were observed in terms of HPL cytokine concentrations, i.e., a clear trend for cytokine deterioration with time, or corresponding MSC proliferation and osteogenic differentiation. Thus, the data herein did not allow for the detection of any statistical associations between specific HPL cytokines and the degree of MSC osteogenic differentiation. Nevertheless, our observation that outdated PCs can be safely frozen for up to 4 months (preferably 3 months when the focus is BTE) may facilitate the implementation of routines enabling more blood banks to produce HPL. This would address the need for standardization and scaling-up of HPL production, while also benefiting blood bank economies.

## Conclusions

The expansion of human MSCs in HPL represents a favorable strategy for BTE. MSCs expanded in HPL demonstrate a high in vitro osteogenic differentiation potential, albeit with considerable donor variation. Exactly which components of HPL contribute to enhancing the osteogenic potential of MSCs is unclear, since HPL contains a complex mixture of cytokines, chemokines, and inflammatory mediators presenting with synergistic effects. Based on the proteomic analysis herein, further investigation of the role of certain cytokines, particularly SCGF, in the regulation of MSCs’ osteogenic differentiation is warranted. Finally, a maximum frozen storage time of 4 months is recommended for outdated PCs assigned for HPL production at blood establishments.

## Supplementary information

**Additional file 1 : Supplementary Table 1**. Real-time PCR assays. **Supplementary Table 2**. Multiplex human cytokine screening panel. **Supplementary Figure 1**. Immunophenotype of BMSCs and ASCs in FBS and HPL. **Supplementary Figure 2**. Cytokine concentrations in HPL.

## Data Availability

Additional data can be made available by the authors upon request.

## Abbreviations

BTE: Bone tissue engineering
MSCs: Mesenchymal stromal cells
BMSCs: Bone marrow-derived mesenchymal stromal cells
ASCs: Adipose tissue-derived mesenchymal stromal cells
SVF: Stromal vascular fraction
GMP: Good manufacturing practice
FBS: Fetal bovine serum
HPL: Human platelet lysate
PCs: Platelet concentrates
qPCR: Quantitative real-time polymerase chain reaction
ELISA: Enzyme-linked immunosorbent assay
ALP: Alkaline phosphatase
GFs: Growth factors
PD: Population doubling rate
PDT: Population doubling time
CPD: Cumulative population doubling
