# Supplementary material for: Influence of platelet storage time on human platelet lysates and platelet lysate-expanded mesenchymal stromal cells for bone tissue engineering
Source: Stem Cell Res Ther. 2020 Sep 23;11:351. doi: 10.1186/s13287-020-01863-9 (PMC7510290; doi:10.1186/s13287-020-01863-9)
Supplement: Supplementary file 1 — Additional file 1 : Supplementary Table 1. Real-time PCR assays. Supplementary Table 2. Multiplex human cytokine screening panel. Supplementary Figure 1. Immunophenotype of BMSCs and ASCs in FBS and HPL. Supplementary Figure 2. Cytokine concentrations in HPL. [file 13287_2020_1863_MOESM1_ESM.docx]

Supplementary table 1: Real-time PCR assays

| **Gene** | **TaqMan^®^ Assay ID** | **Amplicon length** |
| --- | --- | --- |
| GAPDH | Hs 02758991_g1 | 93 |
| RUNX2 | Hs01047973_m1 | 86 |
| BMP2 | Hs00154192_m1 | 60 |
| ALPL | Hs01029144_m1 | 79 |
| COL1A2 | Hs00164099_m1 | 68 |
| SPP1 | Hs00959010_m1 | 84 |
| BGLAP | Hs01587814_g1 | 138 |

*GAPDH* glyceraldehyde 3-phosphate dehydrogenase, *RUNX2* runt-related transcription factor 2, *BMP2* Bone morphogenetic protein 2, *ALPL* alkaline phosphatase, *COL1A2* Collagen type 1, *SPP1* Osteopontin, *BGLAP* Osteocalcin

Supplementary table 2: Multiplex human cytokine screening panel

| **Abbreviation** | **Cytokine** |
| --- | --- |
| b-FGF | Basic fibroblast growth factor |
| Eotaxin/CCL11 | C-C chemokine 11 |
| G-CSF | Granulocyte colony stimulating factor |
| GM-CSF | Granulocyte-macrophage colony-stimulating factor |
| IFN-γ | Interferon-γ |
| IL-1β | Interleukin-1β |
| IL-1ra | Interleukin 1 receptor antagonist |
| IL-1α | Interleukin-1α |
| IL-2Rα | Interleukin-2 receptor α |
| IL-3 | Interleukin-3 |
| IL-12 (p40) | Interleukin-12 subunit beta |
| IL-16 | Interleukin-16 |
| IL-2 | Interleukin-2 |
| IL-4 | Interleukin-4 |
| IL-5 | Interleukin-5 |
| IL-6 | Interleukin-6 |
| IL-7 | Interleukin-7 |
| IL-8 | Interleukin-8 |
| IL-9 | Interleukin-9 |
| GRO-α/CXCL1 | CXC ligand 1 |
| HGF | Hepatocyte growth factor |
| IFN-α2 | Interferon-α2 |
| LIF | Leukemia inhibitory factor |
| MCP-3/CCL7 | Monocyte chemotactic protein-3 |
| IL-10 | Interleukin-10 |
| IL-12 (p70) | Interleukin-12 |
| IL-13 | Interleukin-13 |
| IL-15 | Interleukin-15 |
| IL-17A | Interleukin-17 |
| IL-18 | Interleukin-18 |
| IP-10/CXCL10 | Interferon gamma-induced protein 10/CXC chemokine 10 |
| MCP-1/CCL2 | Monocyte Chemoattractant Protein-1 |
| MIG/CXCL9 | Monokine induced by gamma interferon/CXC ligand 9 |
| β-NGF | Nerve growth factor |
| SCF/KITLG | Stem cell factor/KIT-ligand |
| SCGF-β | Stem cell growth factor |
| SDF-1α/CXCL12 | Stromal cell-derived factor 1 |
| MIP-1α/CCL3 | Macrophage inflammatory protein |
| MIP-1β/CCL4 | Macrophage inflammatory protein |
| PDGF-BB | Platelet-derived growth factor-BB |
| RANTES/CCL5 | Regulated on activation, normal T cell expressed and secreted |
| TNF-α | Tumor Necrosis Factor-α |
| TNF-β | Tumor necrosis factor-β |
| VEGF | Vascular endothelial growth factor |
| CTACK/CCL27 | Cutaneous T-Cell Attracting Chemokine |
| MIF | Macrophage migration inhibitory factor |
| TRAIL | TNF-related apoptosis-inducing ligand |
| M-CSF | Macrophage colony-stimulating factor |

Supplementary Figure 1: Immunophenotype of BMSCs and ASCs in FBS and HPL


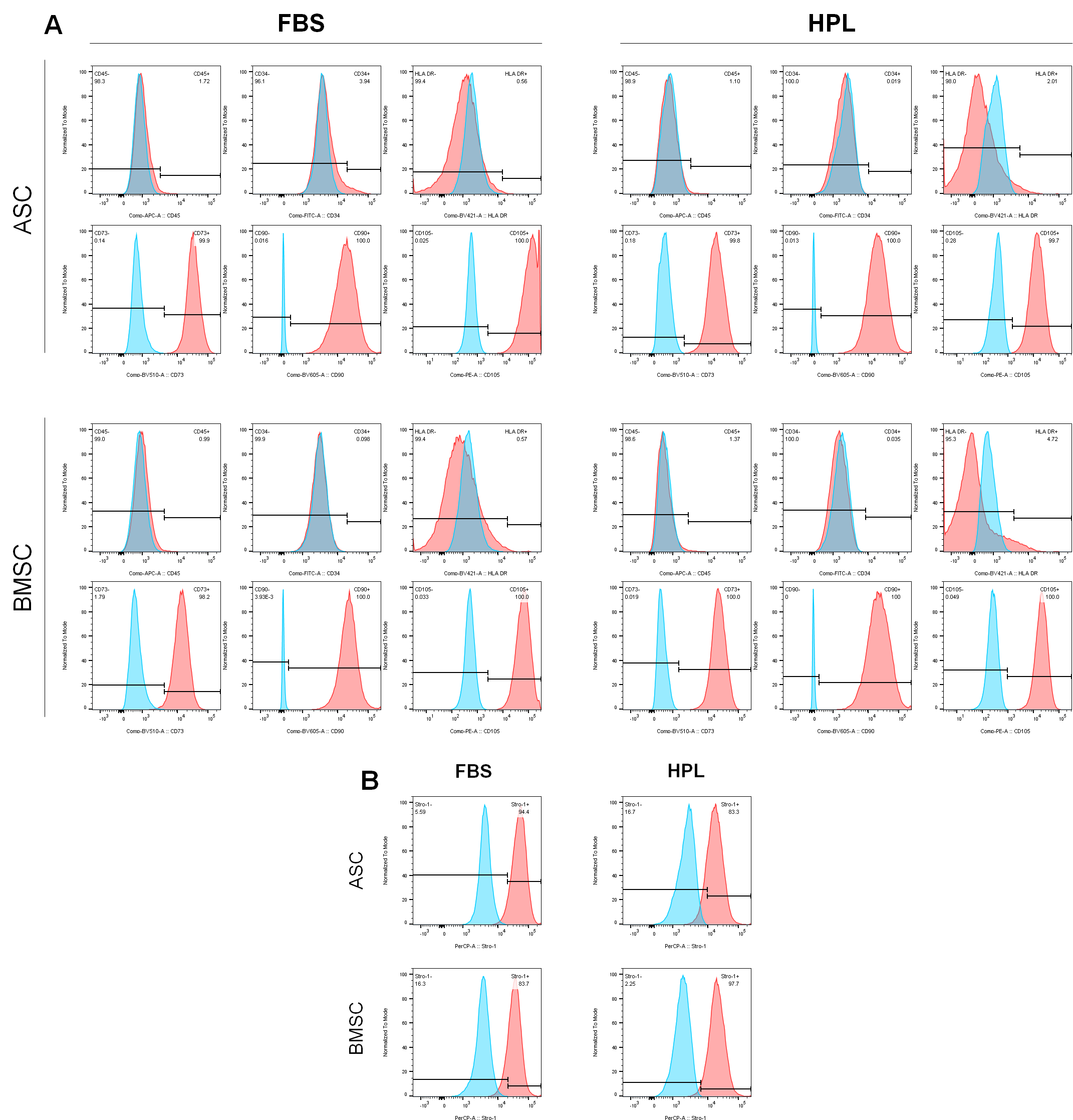


Representative histograms from the flow cytometry analysis of one donor showing surface expression of (A) positive and negative stromal markers, and (B) STRO-1. Expression of stained cells *(red)* and corresponding antibody controls *(blue)*.

Supplementary Figure 2: Cytokine concentrations in HPL


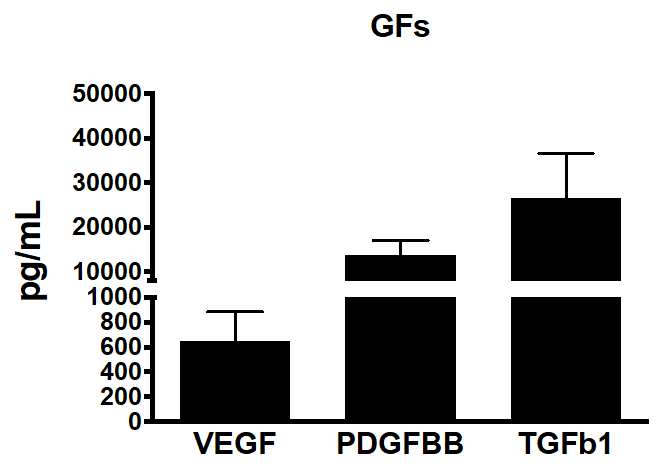


Growth factor (GF) concentrations in HPL determined by ELISA. Data represent means + SD (n=>3 batches)
